# False positive and false negative diagnoses of prostate cancer at multi-parametric prostate MRI in active surveillance

**DOI:** 10.1007/s13244-015-0411-3

**Published:** 2015-05-23

**Authors:** Jeffrey S. Quon, Bardia Moosavi, Maneesh Khanna, Trevor A. Flood, Christopher S. Lim, Nicola Schieda

**Affiliations:** Department of Medical Imaging, The Ottawa Hospital, The University of Ottawa, 1053 Carling Avenue, Ottawa, ON Canada K1Y 4E9; Department of Radiology, Hamad General Hospital, Doha, Qatar; Department of Pathology, The Ottawa Hospital, The University of Ottawa, 501 Smyth Road, Ottawa, ON Canada K1H 8 L6

**Keywords:** Prostate cancer, Active surveillance of prostate cancer, Multi-parametric MRI, Targeted biopsy, Diffusion-weighted imaging

## Abstract

**Abstract:**

MP-MRI is a critical component in active surveillance (AS) of prostate cancer (PCa) because of a high negative predictive value for clinically significant tumours. This review illustrates pitfalls of MP-MRI and how to recognise and avoid them. The anterior fibromuscular stroma and central zone are low signal on T2W-MRI/apparent diffusion coefficient (ADC), resembling PCa. Location, progressive enhancement and low signal on b ≥1000 mm²/s echo-planar images (EPI) are differentiating features. BPH can mimic PCa. Glandular BPH shows increased T2W/ADC signal, cystic change and progressive enhancement; however, stromal BPH resembles transition zone (TZ) PCa. A rounded morphology, low T2 signal capsule and posterior/superior location favour stromal BPH. Acute/chronic prostatitis mimics PCa at MP-MRI, with differentiation mainly on clinical grounds. Visual analysis of diffusion-weighted MRI must include EPI and appropriate windowing of ADC. Quantitative ADC analysis is limited by lack of standardization; the ADC ratio and ADC histogram analysis are alternatives to mean values. DCE lacks standardisation and has limited utility in the TZ, where T2W/DWI are favoured. Targeted TRUS-guided biopsies of MR-detected lesions are challenging. Lesions detected on MP-MRI may not be perfectly targeted with TRUS and this must be considered when faced with a suspicious lesion on MP-MRI and a negative targeted TRUS biopsy histopathological result.

***Keypoints*:**

*• Multi-parametric MRI plays a critical role in prostate cancer active surveillance.*

*• Low T2W signal intensity structures appear dark on ADC, potentially simulating cancer.*

*• Stromal BPH mimics cancer at DWI and DCE.*

*• Long b value trace EPI should be reviewed*

*• Targeted biopsy of MR-detected lesions using TRUS guidance may be challenging.*

## Introduction

Prostate cancer (PCa) is the most common cancer among males in North America [1, 2] and the most common solid neoplasm in Europe [3]. PCa is typically diagnosed with non-targeted systematic trans-rectal ultrasound (TRUS)-guided biopsy in patients with an elevated prostate serum antigen (PSA) level and/or abnormal digital rectal examination (DRE). The management of a particular patient with PCa is multi-factorial and patient specific due to the biological heterogeneity of PCa. Traditional treatment of PCa varies from radical prostatectomy (RP) or radiotherapy (RT) to watchful waiting (delayed symptomatic non-curative treatment of apparently localised PCa in males who are not candidates for aggressive local therapy) [1, 3]. Active surveillance (AS) is defined as the expectant management (deferred immediate therapy) of PCa in carefully selected males with localised disease considered to be at low risk for progression [4]. AS differs from watchful waiting because definitive treatment is used in patients managed with AS when there is evidence that the patient is at an increased risk for disease progression [4, 5]. AS has become the treatment of choice for low-grade, low-volume tumours [6, 7] and is heavily reliant on accurate detection of tumour, accurate estimate of tumour volume and accurate Gleason grading of tumour [6, 7].

Non-targeted TRUS-guided biopsy typically obtains 6–12 core biopsies of the peripheral zone (PZ), which harbours approximately 70 % of cancers [1, 8]. The limitations of non-targeted TRUS-guided biopsy are well known [8] with an estimated 20 % false-negative rate [9–11]. Furthermore, non-targeted TRUS-guided biopsy may yield unreliable information regarding the volume, extent and aggressiveness of PCa; it is has been reported that up to 30–45 % of patients are upgraded/upstaged from their initial diagnosis at TRUS-guided needle biopsy after RP [12]. Moreover, certain areas of the prostate gland [i.e., the anterior gland, transition zone (TZ) and apex] are known to be under-sampled or not sampled at all at routine non-targeted TRUS-guided biopsy and are now increasingly being recognised as areas that may contain clinically significant (CS) tumours [13]. These limitations are of critical importance in AS, where treatment decisions are based on risk stratification and dependent on accurate Gleason grading of tumours [4].

Multi-parametric (MP) MRI [diffusion-weighted imaging (DWI) + dynamic contrast enhancement (DCE) and/or MR spectroscopy] has become the reference standard for prostate imaging endorsed by both the American College of Radiology (ACR) and European Society of Uroradiology (ESUR) [14, 15] (Table 1). Two recent meta-analyses concluded that MP-MRI has a high negative predictive value for the detection of CS cancers [16, 17], and it has been shown previously that MP-MRI can estimate grade of PCa compared to histopathology results with a reasonable degree of accuracy  [18]. Due to the ability of MP-MRI to detect clinically significant (higher volume Gleason score ≥7) tumours with high degrees of accuracy, it has become of tremendous value in AS [19]. Recent studies have demonstrated that MP-MRI can help to determine eligibility for AS [20] and potentially reclassify patients already enrolled in AS before repeat biopsies [21]. In the UK, the National Institute for Health and Care Excellence (NICE) guidelines currently mandate MP-MRI be performed at the onset of AS protocols and that MP-MRI be performed in patients while they are enrolled in AS when there is concern about clinical or PSA changes [22].

**Table 1:** Sequence parameters for multi-parametric MRI of the prostate protocol performed with a pelvic surface coil^a^ at 3 T^b^

	Imaging plane	Field of view (mm)	Matrix size	Slice thickness/gap (mm)	TR/TE (ms)	Echo train length	Flip angle	Acceler-ation factor	Receiver bandwidth (Hz/voxel)	Acquisition time (min)	Number of signals averaged
T1 TSE^c^	Axial	350 × 350	320 × 320	5.0/1.0	720/8–14	3	111	N/A	244	4 min	2
T1 3D dual-echo GRE^d^	Axial	240 × 240	292 × 224	4.0/1.0	4.8/1.1–1.3;TE12.2–2.5; TE2	N/A	12	2	558	Breath hold	1
T2 TSE	Coronal Sagittal Axial	220 × 220	320 × 256	4.0/0 3.0/0 3.0/0	3890–5250/105–125	27–35	111	N/A	122	4 min 4 min 4 min	1–2
DWI^e^	Axial	280 × 280	128 × 80	5.0/1.0	4200/90	1	90	2	1950	5 min	4–10
T1 GRE^f^ dynamic contrast	Axial	220 × 220	128 × 128	4.0/0	4.3/1.3	N/A	12	2	488	6 min	1

^a^Integrated pelvic surface coils (4–16 channels) with activated spine coils (8–12 channels)

^b^Clinical 3-T systems: TRIO TIM (Siemens Medical, Malvern, PA) and Discovery 750 W (General Electric, Milwaukee, WI)

^c^Turbo/fast spin echo

^d^Gradient recalled echo

^e^ *DWI* Diffusion-weighted imaging performed with spectral fat suppression echo planar imaging with tridirectional motion probing gradients and *b* values of 0, 0, 500, 1000, 1500 mm²/s with automatic apparent diffusion coefficient map generation

^f^Dynamic fast spoiled 2D GRE performed with a temporal resolution of 10 s after injection of 0.2 mmol/kg of gadobutrol (Gadovist, Bayer Inc., Toronto, ON) at a rate of 3 ml/s

A variety of interpretive and technical pitfalls may be encountered at MP-MRI of the prostate. A failure to recognise and correct these errors in AS patients can result in suboptimal care. False-positive diagnoses of areas of potential cancers at MP-MRI create clinical uncertainty and often lead to multiple unnecessary biopsies or in certain cases surgical management of low-grade, low-volume disease. Moreover, a failure to recognise clinically significant cancers in males being considered for or treated with AS could result in suboptimal patient outcomes. The purpose of this review is to illustrate both interpretive and technical pitfalls encountered at MP-MRI in the active surveillance population and how to detect, correct and avoid them.

## Interpretive pitfalls

1. **Normal anatomic structures can mimic anterior and TZ cancers**

**Fig. 1:**
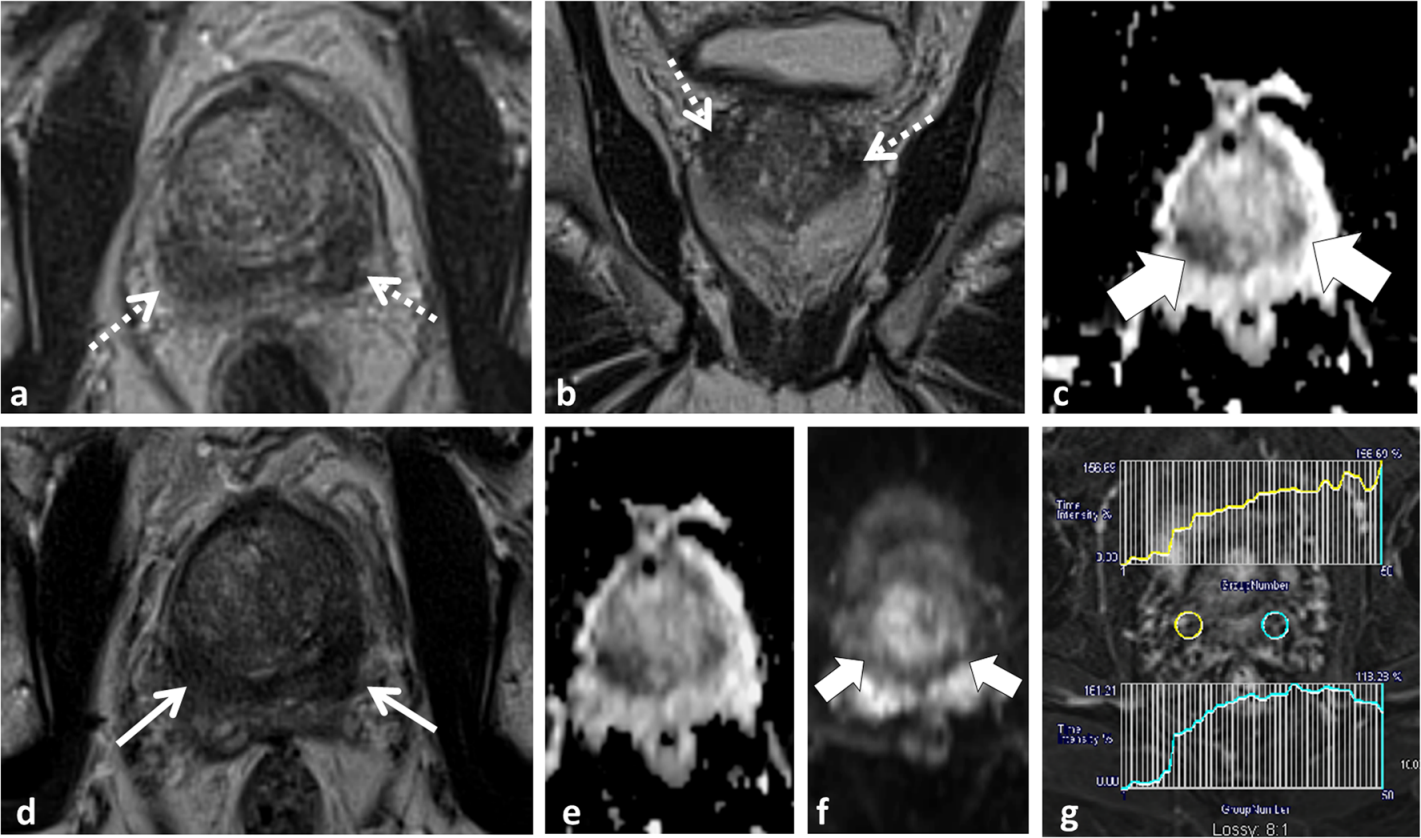
A 54-year-old male with low volume Gleason score 3 + 3 = 6 PCa at TRUS-guided biopsy in the right middle peripheral zone (*PZ*). MP-MRI was performed because of rising PSA to exclude clinically significant (*CS*) tumour. Axial (**a**) and coronal (**b**) T2-weighted (T2W) turbo spin echo (*TSE*) images demonstrate bilateral foci of low T2 signal intensity (SI) at the prostate base (*dotted arrows*) adjacent to the insertion of the vas deferens (not shown). Axial apparent diffusion coefficient (*ADC*) map (**c**) at the same level demonstrates corresponding low SI (*white arrows*). These areas were described as suspicious and repeat targeted TRUS-guided biopsy was suggested. A repeat biopsy with multiple cores through both areas yielded only normal prostatic tissue. A repeat MRI performed 2 years later demonstrates similar findings on the T2W (**d**) and ADC map (**e**), which represent the normal central zone. Corresponding b1000 mm²/s trace echo-planar image (EPI) does not show concordant areas of increased SI (*white arrows* in **f**) and there is benign progressive enhancement on dynamic contrast-enhanced (DCE) imaging (**g**)

A detailed understanding of the normal zonal anatomy of the prostate is essential for interpretation of prostate MRI. In 1981, McNeal [23] described the three distinct prostate zonal regions: (1) the peripheral zone, (2) transition zone and (3) central zone (CZ). The prostatic zonal anatomy is best depicted at T2-weighted (W) MRI (Fig. 1). The PZ is hyperintense on T2W because of abundant glandular tissue, is located at the periphery of the gland and harbours 70 % of PCa [24] (Fig. 1). Previously, it was thought that the TZ [the site of benign prostatic hyperplasia (BPH)] and the CZ (which surrounds the ejaculatory ducts, is located mainly at the base of the prostate, is posterior to the TZ and the urethra, and is proximal to the verumontanum) could not be differentiated at imaging and these areas were collectively referred to as the central gland [25, 26]. The central gland in most adult males consists of hypertrophied TZ that compresses the CZ against the surgical capsule [25]. Currently, it is acknowledged that the CZ can be identified separately from the TZ in up to 4/5 of males and the CZ appears as a symmetric band of homogeneously low signal intensity (SI) on T2W MRI and apparent diffusion coefficient (ADC) maps best seen at the prostate base [25], (Fig. 1).

**Fig. 2:**
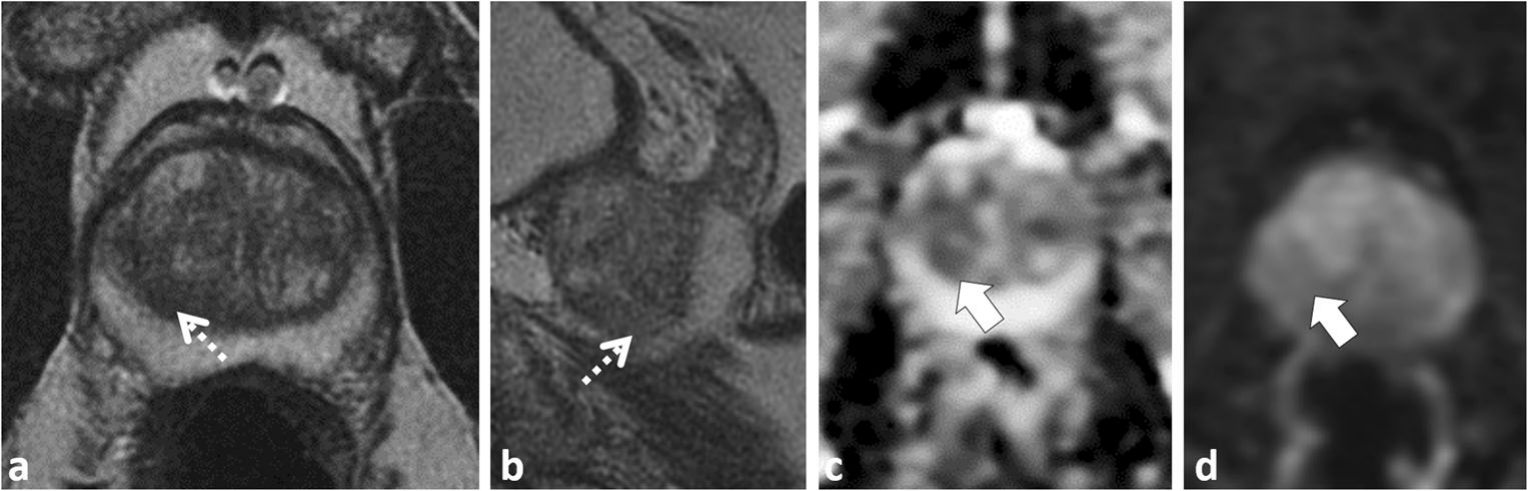
A 51-year-old patient with low-volume Gleason score 3 + 3 = 6 at non-targeted TRUS-guided biopsy. MP-MRI was performed at onset of active surveillance (AS) to exclude higher grade tumour. Axial (**a**) and sagittal (**b**) T2W images demonstrate an ill-defined ovoid lesion of low T2 SI at the junction of the right PZ and transition zone (TZ) (dotted *white arrow*) along the course of the surgical capsule. Axial ADC map image (**c**) at the same level demonstrates corresponding low SI (*white arrow*). This area was considered suspicious for tumour. Targeted TRUS biopsy was performed and yielded only normal prostate tissue. The area did not change on follow-up MP-MRI and represents asymmetry of the normal central zone. In retrospect, corresponding b1000 mm²/s trace EPI (**d**) did not show increased signal (*white arrow*) in this area

Accurate identification of the CZ is critical as, in our experience, many false-positive interpretations of PCa occur when mistaking the CZ for PCa. The CZ can be diagnosed by noting its symmetry (particularly on coronal T2W images) and typical location adjacent to the ejaculatory ducts [25, 26]. Functional imaging techniques also aid in the differentiation of CZ from PCa. In the normal CZ, low SI on ADC is due to inherently low T2 SI not true restricted diffusion (“T2 black hole effect”); see Technical Pitfall 2. In the normal CZ, there is no true diffusion restriction; therefore, there should be no increased SI on long (b ≥1000 mm²/s) EPI (Figs. 1 and 2). The enhancement pattern of the CZ at DCE has not, to our knowledge, been described; however, in our experience, a progressive type 1 enhancement is typical of the CZ (Fig. 1). CZ PCas are aggressive [26–28], but account for less than 5 % of PCa [26]. An asymmetry of the central zone should raise suspicion for a possible tumour [25]; however, it is more common to observe a slight asymmetry in the thickness of the CZ because of the orientation of the prostate gland and the plane of imaging (Fig. 2). To diagnose the rare CZ PCa, asymmetry at T2W should be confirmed in multiple planes and corresponding functional imaging findings such as increased signal intensity on long b value trace EPIs and/or suspicious contrast kinetics at DCE are helpful, as these latter findings are not expected in the normal CZ (Fig. 2). Ultimately targeted biopsy or MRI follow-up may be necessary for suspicious areas in the CZ.

**Fig. 3:**
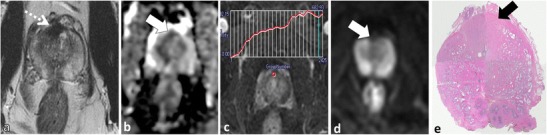
A 54-year-old patient with elevated and rising PSA. Axial T2W TSE (**a**) demonstrates a low SI focus in the anterior midline (*dotted arrow*) with corresponding low SI (*white arrow*) on the axial ADC map (**b**). This area was reported as suspicious for tumour. Repeat non-targeted biopsies of the PZ revealed Gleason score 3 + 4 = 7 tumour in the right PZ (not shown) and the patient underwent radical prostatectomy (RP). The structure highlighted on MRI is the normal anterior fibromuscular stroma (AFMS); note: characteristic midline anterior location on axial T2 (**a**) and benign progressive enhancement on DCE (**c**). Also note that low SI on the ADC (**b**) map is due to inherently low T2 SI rather than restricted diffusion; there is a lack of increased SI on trace b1000 mm²/s EPI (**d**). Whole-mount histopathology image of the RP specimen (**e**) demonstrates the AFMS (*black arrow*), which is predominantly composed of smooth muscle that blends with the overlying prostate stroma

**Fig. 4:**
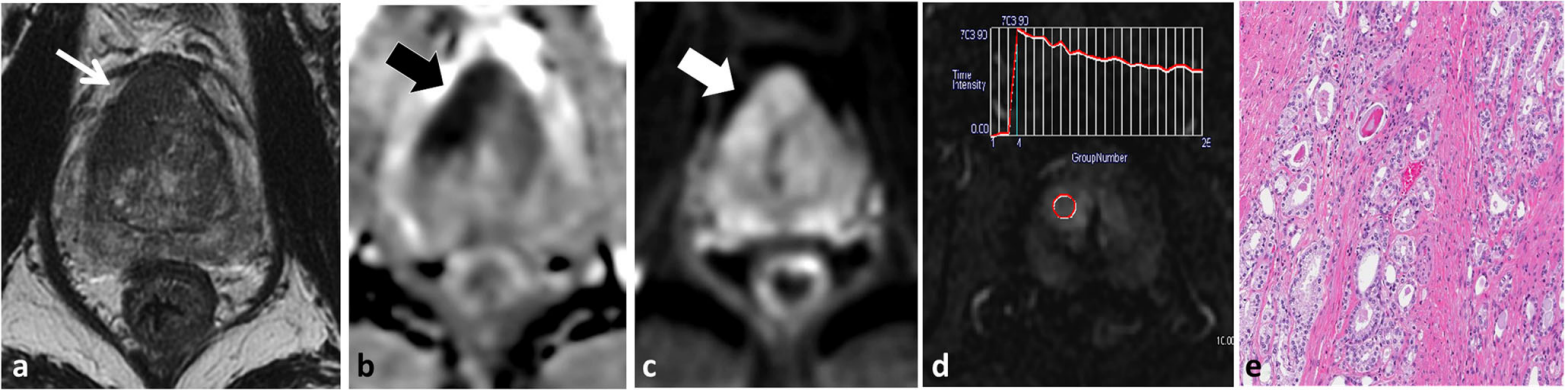
A 50-year-old patient with rising PSA and a typical anterior tumour involving the right side of the gland. Axial T2W TSE (**a**) demonstrates an amorphous, off midline, lenticular-shaped, ill-defined low T2 SI region (*white arrow*) with low SI (*black arrow*) on the ADC (**b**) map due to restricted diffusion (note increased SI on trace b1000 mm²/s EPI (*white arrow* in **c**) and type III contrast curve kinetics on DCE (**d**). A targeted TRUS biopsy was performed and confirmed Gleason score 4 + 3 = 7 tumour anteriorly. Corresponding microscopic image from TRUS biopsy (**e**) demonstrates Gleason pattern 4 tumour

The anterior fibromuscular stroma (AFMS) is a normal structure at the extreme anterior midline of the prostate gland, which is inseparable from the surrounding normal prostatic stroma [25, 29]. The AFMS contains no glandular tissue and is composed of widely spaced smooth muscle cells. It appears homogeneously hypointense on T2W MRI and because of this inherent low T2W SI will appear as low SI on ADC maps. Differentiation of the AFMS from anterior cancer is established by noting the midline location, well-defined margins, lack of true diffusion restriction (absence of high SI on b ≥1000 mm²/s EPI) and a benign progressive type 1 enhancement pattern at DCE (Fig. 3) [25, 30]. PCa arising in the extreme anterior prostate can resemble the AFMS and in many cases anterior tumors can extend up to or invade the AFMS. Both lesions are low SI on T2W; however, anterior PCa is more lenticular or polygonal in shape, has ill-defined or smudged borders, and the bulk of the lesion tends to be off midline (Fig. 4) [31]. The use of functional imaging sequences is contributory (Fig. 4) [30]. DWI increases accuracy for detection of anterior cancers because anterior tumours will be of low SI on ADC because of true restricted diffusion, demonstrating increased SI on trace b ≥1000 mm²/s EPI [32]. The utility of DCE in the TZ and anterior prostate is controversial [33]; however, authors have demonstrated more aggressive enhancement patterns in anterior PCa [34–37]. In our experience, DCE can be beneficial to discriminate anterior PCa from AFMS; however, overlap with stromal BPH limits its utility in the TZ (discussed later) [35].

(2) **Post-biopsy haemorrhage can mimic PZ PCa on T2W MRI**

**Fig. 5:**
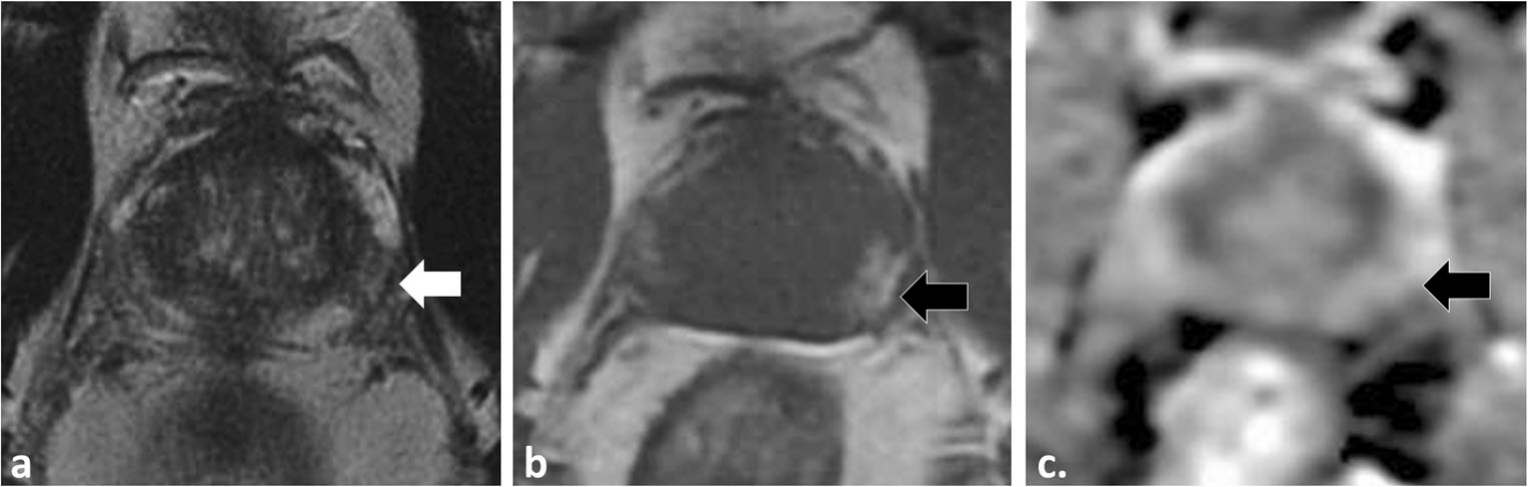
A 58-year-old male patient with Gleason score 3 + 3 = 6 tumour imaged with MP-MRI because of rising PSA. On axial T2W TSE (**a**) there is a low SI focus in the left mid PZ (*white arrow*), which could represent peripheral zone cancer. Corresponding axial T1W TSE (**b**) demonstrates increased T1 SI (*black arrow*) in keeping with post-biopsy haemorrhage. Axial ADC map (**c**) does not reveal the lesion (*black arrow*), which was readily diagnosed as post-biopsy haemorrhage

Detection of PCa on T2W is predicated on the ability to discriminate low T2 SI tumour from the normal adjacent high T2 SI PZ [15, 38–40]. The glandular tissue of the PZ contains high concentrations of citrate, which is a natural anti-coagulant; therefore, post-procedural haemorrhage is commonly observed at MRI after prostate biopsies and can persist for up to 4 months after the procedure [41]. Areas of post-biopsy haemorrhage are characteristically hypointense on T2W MRI in up to 80 % of cases and can mimic PCa [41] (Fig. 5). This pitfall is easily avoided by cross-referencing T2W to pre-contrast T1W imaging because areas of haemorrhage will also be characteristically hyperintense on T1W (Fig. 5).

Many institutions continue to defer prostate MRI studies until 3–8 weeks have passed after the biopsy to reduce the amount of post-biopsy haemorrhage and facilitate interpretation. With the advent of functional imaging sequences post-biopsy haemorrhage is, in our experience, much less problematic. Areas of tumour have been shown to be reliably differentiated from haemorrhage using DWI, and DCE analysis can be interpreted through image subtraction [42] (Fig. 5). Moreover, because PCa contains less citrate than the normal PZ, areas that are devoid of haemorrhage in the PZ after biopsy may actually highlight the tumour on T1W imaging (“haemorrhage exclusion sign”) [42]. This imaging finding has recently been shown to have >95 % positive predictive value for tumour localisation when combined with characteristic imaging findings at T2W MRI [42].

(3) **Benign prostatic hyperplasia (BPH) resembles TZ PCa**

BPH is extremely common in the TZ and its prevalence increases with age. BPH can be nodular and TZ nodules are commonly encountered at MP-MRI [35], and nodular BPH may mimic TZ PCa [25]. Nodular BPH is categorised into three main subtypes: glandular, stromal and mixed.

**Fig. 6:**
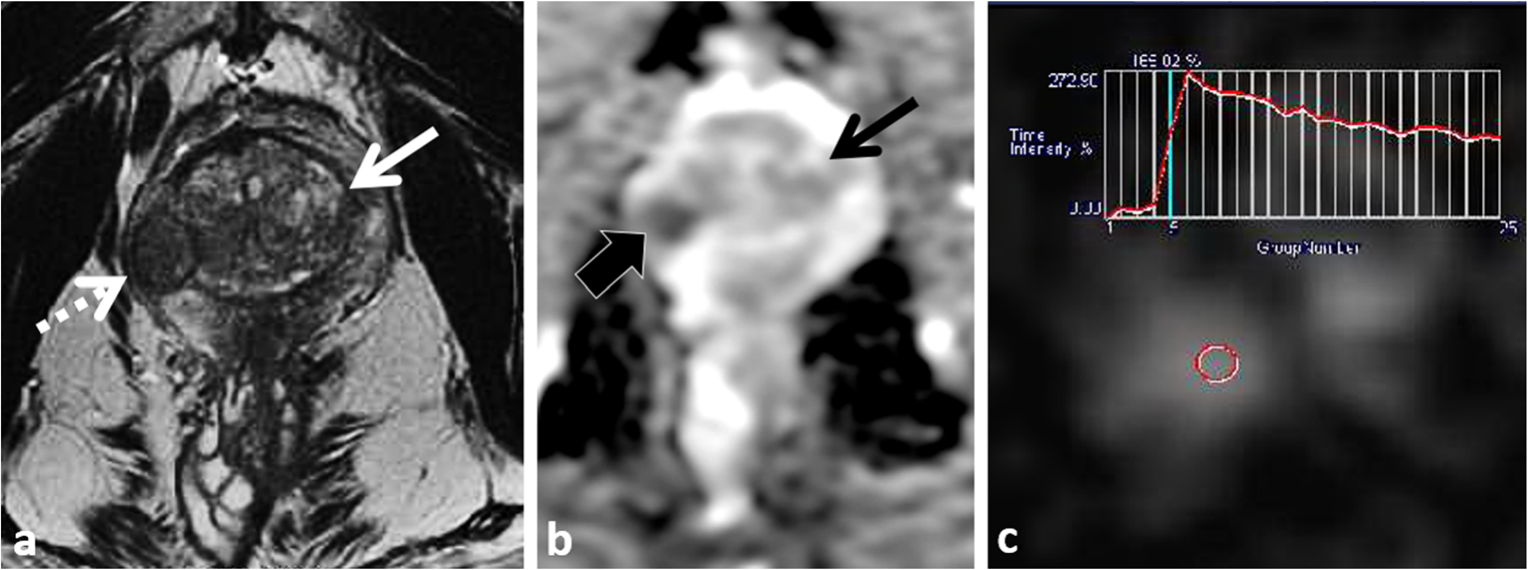
A 47-year-old patient post-total colectomy for ulcerative colitis and elevated PSA. MP-MRI was performed to evaluate for potential tumour and to plan a biopsy. Axial T2W TSE (**a**) image demonstrates enlargement of the central gland (TZ) consistent with BPH. There is a well-circumscribed, round, mixed but predominantly increased T2W SI nodule with internal cystic change (*white arrow*) and a predominantly homogeneously low T2 SI nodule at the junction of the right middle PZ and central gland (*dotted arrow*). The larger nodule demonstrates T2 shine-through on the ADC map (*black arrow* in **b**) and is characteristic of glandular BPH. The other nodule demonstrates restricted diffusion (*thick black arrow* in **b**) and type III kinetics at DCE (**c**). A diagnosis of prostate cancer was suggested. Targeted biopsy revealed normal prostatic tissue and stromal BPH. Follow-up MRI (not shown) demonstrated no change and the PSA was stable. In retrospect, the nodule is round, well-circumscribed and demonstrates a complete low T2 SI rim (**a**), findings that are more in keeping with stromal BPH rather than cancer

Glandular BPH is characterised histologically by hyperplasia of glandular tissue with papillary buds, infoldings and cysts. Glandular BPH is readily differentiated from TZ PCa at MP-MRI [35] and typical features include a well-circumscribed, sharply demarcated and rounded shape, a continuous low T2W SI rim, increased T2W SI often with cystic change, low SI on long ≥1000 mm²/s *b* value EPI, “T2 shine-through” on ADC maps and benign/progressive/type I contrast kinetics with a low transfer constant on DCE (Fig. 6) [25, 35].

**Fig. 7:**
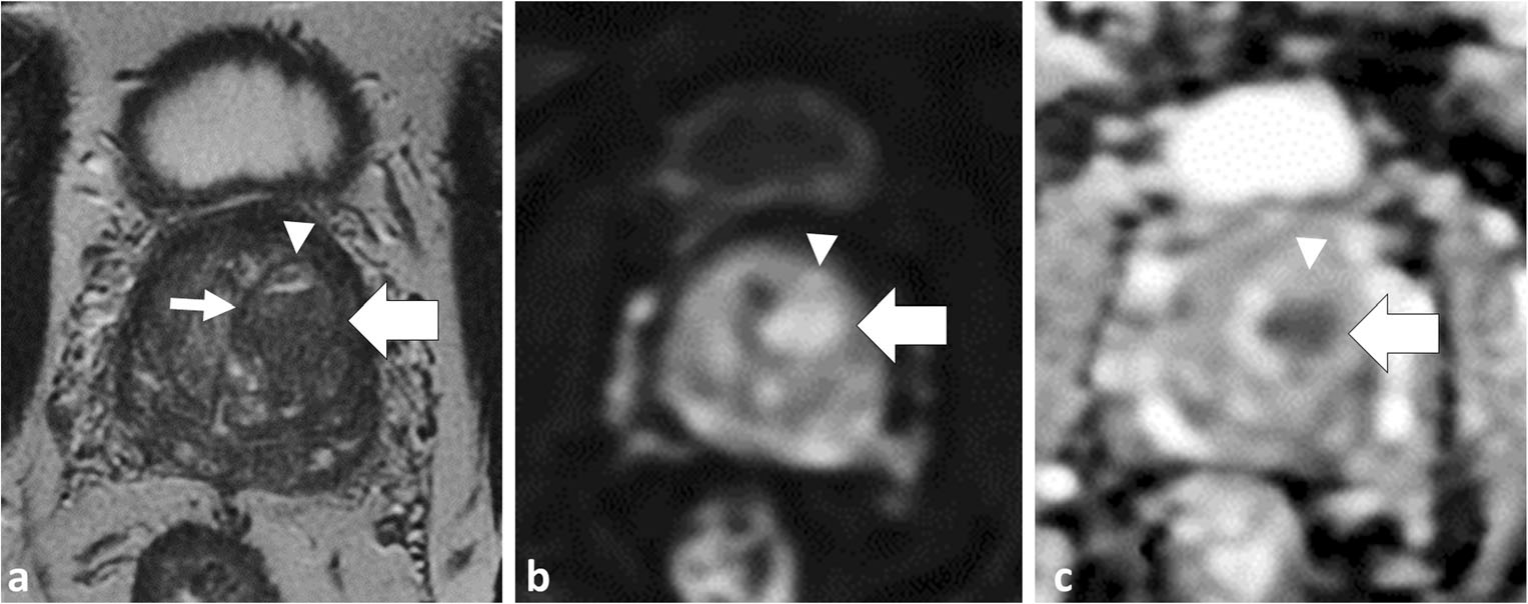
A 58-year-old patient with low-volume, low Gleason score 3 + 3 = 6 tumour in the right mid-peripheral zone undergoing MRI prior to repeat biopsy because of rising PSA. Axial T2W TSE (**a**) image demonstrates a low T2 SI nodule in the left TZ (*thick white arrow*). The nodule is predominantly homogeneously of low T2W SI but demonstrates a small focus of cystic change anteriorly (*arrowhead*). Note that the nodule is round and demonstrates a continuous low T2W SI rim (*thin white arrow*). The nodule demonstrates restricted diffusion, increased SI on trace b1000 mm²/s EPI (**b**) and low SI on the ADC map (**c**) (*thick white arrows*). Note that cystic change demonstrates T2 shine-through (*arrowhead*). Imaging findings are characteristic of a mixed but predominantly stromal BPH nodule

The presence of increased smooth muscle, lymphocytes and ducts (not associated with prostatitis) and reduced elastic tissue characterises stromal BPH at histopathology. Stromal BPH nodules can be difficult to differentiate from TZ PCa [35] (Figs. 6 and 7). Stromal BPH is typically of low T2W SI, similar to TZ PCa. Stromal BPH is also of low signal intensity on ADC maps because of both inherently low T2W SI and true restricted diffusion (due to compact cellularity) and may or may not have increased SI on long b ≥1000 mm²/s trace EPI. Furthermore, ADC values in stromal BPH are low and overlap with TZ PCa [25, 35]. DCE is limited to differentiate stromal BPH from TZ PCa, because enhancement patterns of stromal BPH overlap with TZ PCa [33–35]. Previous authors have reported lower ADC and higher K-trans values in TZ PCa compared to stromal BPH, but substantial overlaps exist (Figs. 6 and 7) [35]. Other differentiating features that are more suggestive of stromal BPH include well-defined sharply demarcated margins, a continuous low T2W SI rim and a rounded shape (Figs. 6 and 7) [31, 33].

At histopathology and MP-MRI, it is more common to identify a TZ BPH nodule with features of both glandular and stromal BPH. Typically one pattern (glandular or stromal) will predominate; however, features of both types of BPH can be seen within the same nodule, which can further complicate the diagnosis. In cases of mixed BPH, a combination of imaging features of glandular and stromal BPH are encountered [31]. Usually, the presence of increased T2W SI and cystic change (from glandular BPH) within a lesion are reassuring findings of BPH (Fig. 7) since these findings are rarely encountered in PCa.

Chesnais et al. previously demonstrated that the majority of TZ cancers involve the anterior and apical third of the gland [43]. At histopathology, Bouye et al. previously demonstrated that anterior tumours tend to grow anteriorly and invade the anterior fibromuscular stroma but rarely extend to the posterior PZ [44]. It should be noted that in some instances, when there is uncertainty at MP-MRI regarding the diagnosis of TZ PCa versus stromal BPH due to overlap in imaging features, a targeted biopsy or follow-up MP-MRI may be suggested (Fig. 6).

(4) **Acute and chronic prostatitis mimics PCa**

**Fig. 8:**
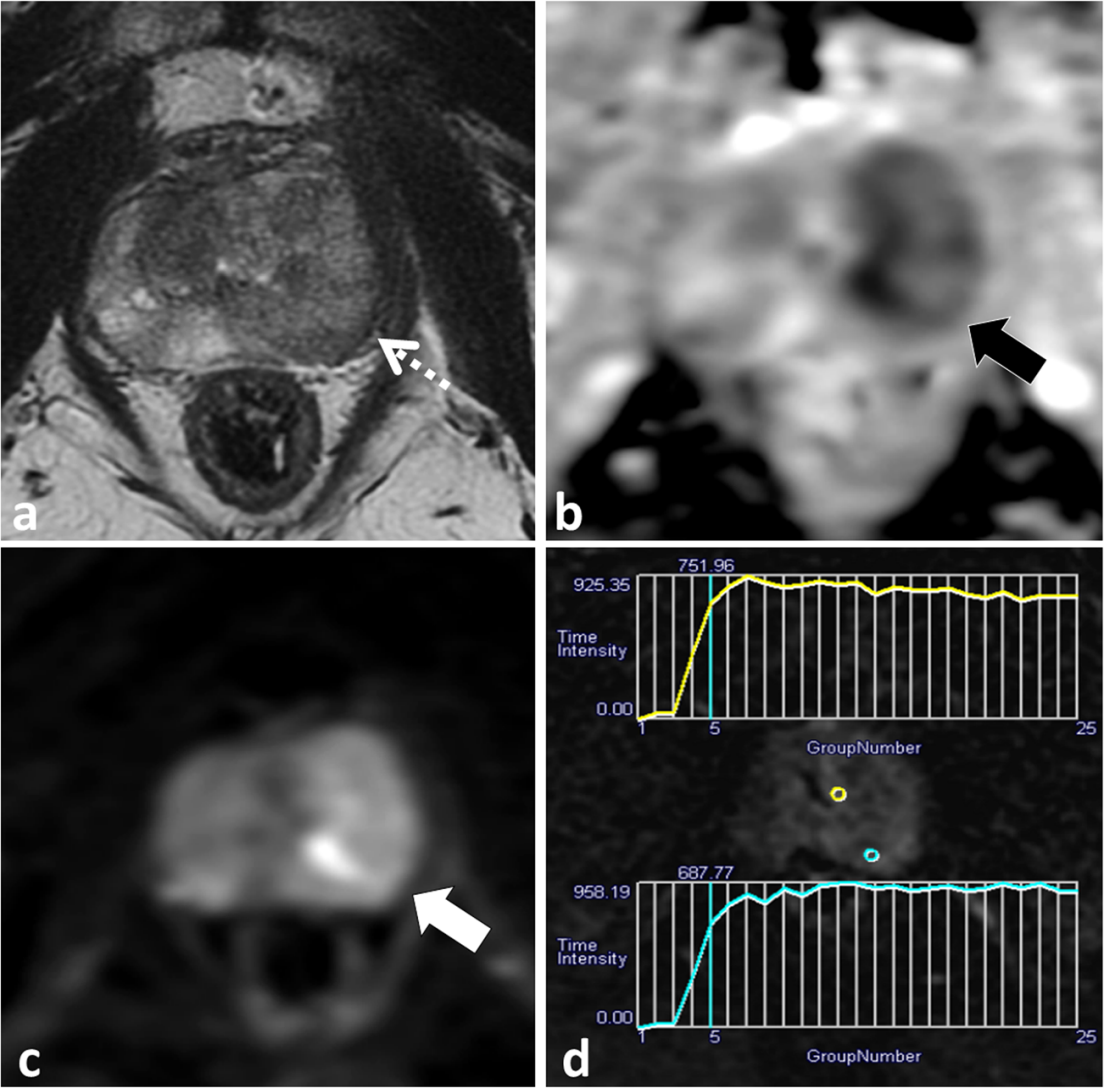
Acute prostatitis in a 46-year-old patient with elevated PSA. Axial T2W TSE (**a**) demonstrates an amorphous area of low T2 SI (*dotted arrow*) in the left middle PZ with corresponding low SI on ADC map (*black arrow* in **b**) due to restricted diffusion, note increased SI on trace b1000 mm²/s EPI (*white arrow* in **c**). There is an indeterminate type II contrast curve (plateau kinetics) on DCE in (**d**). A differential diagnosis of prostatitis or cancer was provided, and biopsy or follow-up was suggested. The patient had typical findings of acute bacterial prostatitis clinically and was treated with antibiotics with normalisation of PSA post therapy

Acute bacterial prostatitis (ABP) is commonly encountered in clinical practice and often managed conservatively without imaging or surgical intervention [38]. When patients with ABP are imaged in the acute setting, prostatitis can mimic PCa at MP-MRI, demonstrating low SI on T2WI, restricted diffusion [45] and enhancement patterns that overlap with PCa [25] (Fig. 8). Although PCa has been reported to demonstrate more well-defined borders and nodular appearance compared to prostatitis and lower ADC values [45, 46], aside from the presence of abscess formation in prostatitis, differentiation is mainly based on clinical grounds.

**Fig. 9:**
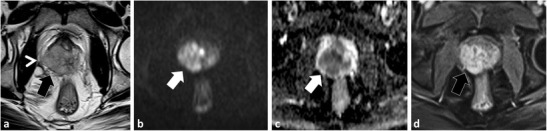
A 64-year-old patient with rising PSA and history of recurrent urothelial cell carcinoma of the urinary bladder treated with intra-vesical BCG therapy. Axial T2W image (**a**) demonstrates a large mass infiltrating throughout the right prostate (*black arrow*) and breaching the prostate capsule laterally (*arrowhead*) consistent with extra-prostatic spread. Axial b1000 mm²/s (**b**) and ADC map (**c**) demonstrate marked restricted diffusion (*white arrows*) and axial image from DCE shows marked early mass-like hyper-enhancement (type III curve was depicted and is not shown). TRUS biopsy was performed and histology was compatible with diffuse granulomatous prostatitis

Granulomatous prostatitis (GP) is a benign inflammatory entity that may also be indistinguishable from PCa at MP-MRI, demonstrating low SI on T2W, restricted diffusion and suspicious enhancement at DCE [47]. Moreover, granulomatous prostatitis can involve the periprostatic fat or seminal vesicles, mimicking extraprostatic spread of PCa (Fig. 9). With the correct clinical history [e.g., previous bacille Calmette-Guerin therapy for bladder cancer, tuberculous prostatitis or previous intervention such as transurethral resection of the prostate (TURP)], granulomatous prostatitis may be favoured over PCa [25]; however, histological confirmation is often required [47].

**Fig. 10:**
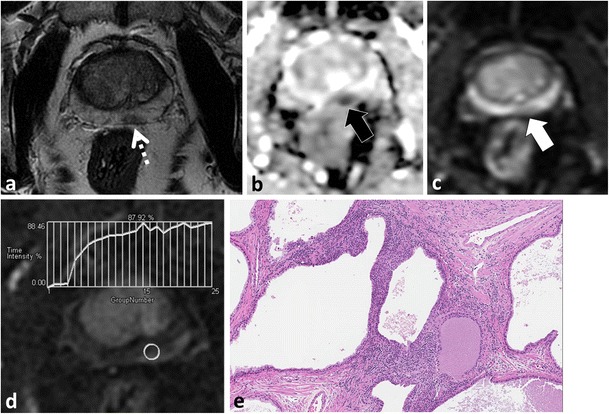
A 64-year-old patient with Gleason score 3 + 3 = 6 in <5 % of one core in the left middle PZ underwent MRI prior to enrolment into active surveillance. Axial T2W TSE image (**a**) demonstrates a low T2 SI focus in the left PZ (*dotted arrow*) with low SI on ADC (*black arrow* in **b**). Based on these findings a diagnosis of potential higher grade tumour was suggested and a repeat biopsy was performed. Saturation biopsies through the left mid PZ revealed only normal prostatic tissue and areas of chronic prostatitis. In retrospect, trace b1000 mm²/s EPI demonstrates low SI (*white arrow* in **c**), which indicates that there is no restricted diffusion in this area. Similarly, there is a benign progressive type I enhancement curve on DCE (**d**), which further argues against a higher grade tumour. Corresponding histopathology slide (**e**) demonstrates areas of chronic inflammation

In the chronic setting, areas of prostatitis can mimic PCa because they will demonstrate low T2W SI [48]. Functional imaging techniques can be useful to differentiate areas of chronic prostatitis from cancer (Fig. 10) but there is overlap in imaging features [49, 50] and targeted biopsy or MP-MRI follow-up of the suspicion region may be considered depending on the level of suspicion for tumour [47].

(5) **Ductal variant adenocarcinoma may be occult on T2W MRI**

**Fig. 11:**
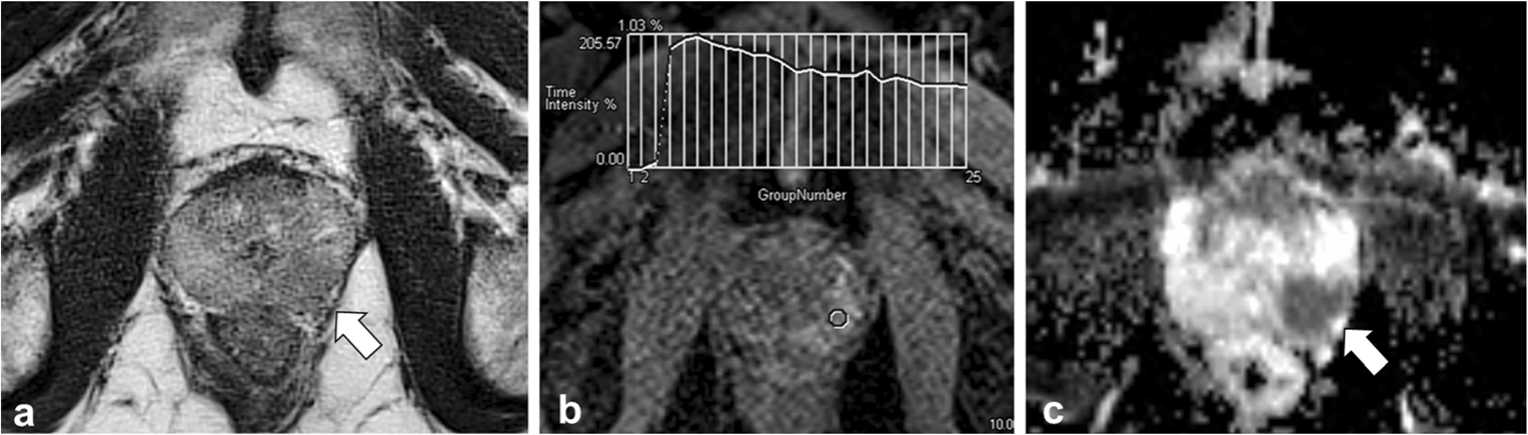
A 59-year-old patient with Gleason 3 + 4 = 7 cancer in the left middle PZ on TRUS biopsy underwent MP-MRI for staging. Axial T2W TSE image shows a subtle lesion in the left middle PZ (*white arrow* in **b**) with type III kinetics on DCE (**b**) and profound restricted diffusion on ADC map (*white arrow* in **c**). Typically with conventional acinar adenocarcinoma T2W signal intensity mirrors findings on ADC map, the discordant findings with only minimally decreased T2W signal and profound restricted diffusion can be seen in ductal variant adenocarcinoma. Final histopathology after RP was Gleason score 4 + 4 = 8 tumour with dominant ductal component

PCa is divided into the most common acinar adenocarcinoma and less common non-acinar subtypes. Ductal adenocarcinoma (DCa) is an aggressive variant of PCa and is the most common of the non-acinar subtypes [51] with a reported incidence of 0.5–6 % [52]. DCa is associated with higher rates of positive surgical margins, extraprostatic extension, vascular invasion, seminal vesicle invasion and metastases [53, 54]. A diagnosis of DCa at needle biopsy requires definitive therapy and is considered an absolute contraindication for AS [55, 56]. Schieda et al. recently demonstrated that DCa is of increased SI on T2W MRI, which can render the tumour occult [39]; however, the same authors demonstrated that DCa resembles Gleason score ≥7 or higher tumour on DWI and DCE [57]. Therefore, in a lesion with aggressive imaging findings on DWI/DCE and paradoxical increased T2W SI, DCa should be considered [57] (Fig. 11).

## Technical pitfalls

1. **T2W motion correction with radial acquisition obscures some PCa**

**Fig. 12:**
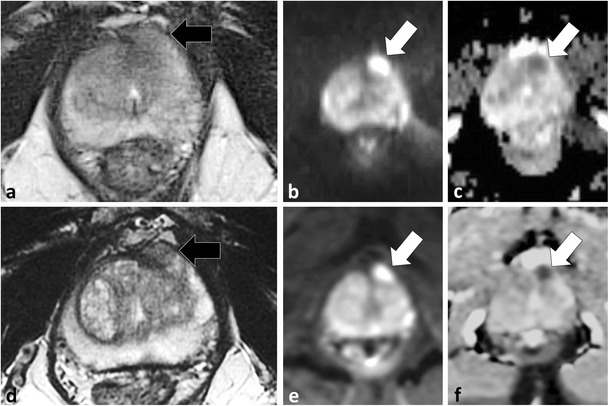
A 63-year-old patient with elevated PSA and previously negative non-targeted TRUS-guided biopsy with persistent clinical suspicion of prostate cancer underwent MP-MRI in two separate sessions within 3 months demonstrating the loss of contrast with BLADE/PROPELLER imaging. Axial T2W BLADE **a**, b1000 mm²/s EPI **b** and ADC map **c** demonstrate a suspicious focus of restricted diffusion in the left middle anterior horn of the PZ (*white arrows*), which is not visible on axial T2W BLADE (*black arrow* in **a**). Repeat examination performed using T2W FSE (**d**) reveals a T2 hypointense nodule in the same location (*black arrow* in **d**) with persistent restricted diffusion (*white arrows* in **e** and **f**)

As discussed, detection of tumour in the PZ on T2W MRI is based on differentiation of low T2W SI tumour from the high T2W glandular tissue [15, 38–40]. In the TZ, T2W imaging is now recognised as the most important pulse sequence for detection of PCa because of overlap between TZ cancer and stromal BPH on functional imaging (see Interpretive pitfalls, stromal BPH) [58]. T2W imaging is typically performed using turbo/fast spin-echo techniques [39]. Motion correction with radial acquisition (BLADE, Siemens Healthcare, Malvern, PA, USA; PROPELLER, General Electric Healthcare, Milwaukee, WI, USA) sequences are popular for pelvic imaging because they correct for in-plane rotation and translational artefacts and have been shown to result in improved overall image quality when compared to conventional spin echo [59, 60]. A disadvantage of these sequences is decreased image contrast compared to conventional spin echo [59]. Recently, Rosenkrantz et al. demonstrated that a minority of PCa foci may be obscured at T2W MRI when BLADE/PROPELLER is used compared to conventional spin echo [61] (Fig. 12).

(2) **Visual/quantitative analysis of DWI for tumour detection/grading is complex**

**Fig. 13:**
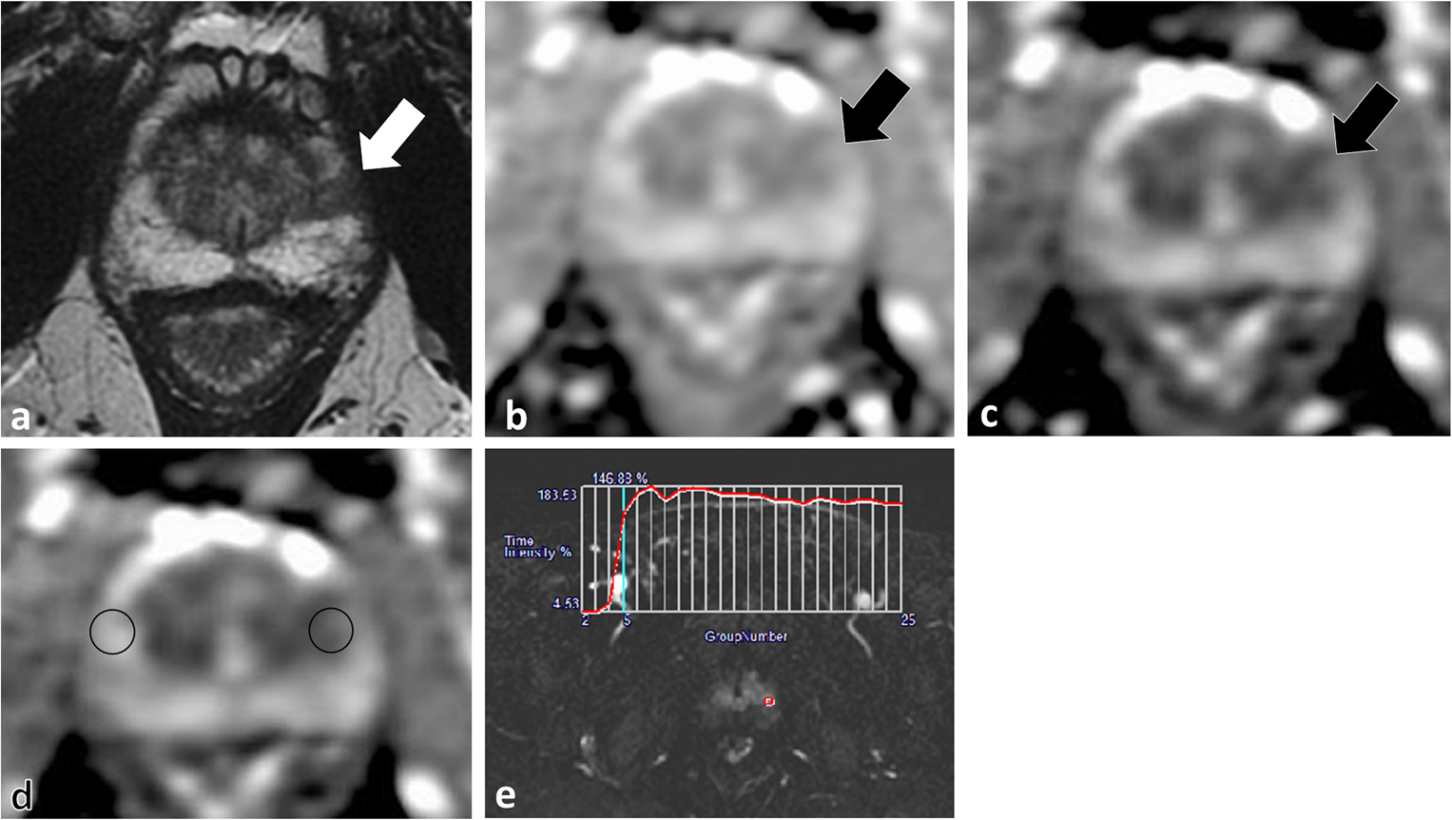
A 55-year-old patient with low-volume Gleason score 3 + 3 = 6 tumour at TRUS biopsy in the left apical PZ underwent MRI prior to consideration for potential AS. Axial T2W TSE image (**a**) demonstrates a low T2 SI focus in the left apical PZ (*white arrow*). Axial ADC map displayed with automatic windowing/levelling shows minimal decreased SI (*black arrow* in **b**). These findings could be considered typical for a 3 + 3 = 6 cancer. With modified display of the ADC map using previously validated settings (width = 1.650 and level = 1.675 × 10–6 mm²/s) the nodule (*black arrow* in **c**) is noted to be of lower SI than initially displayed in (**b**). Using quantitative data, the ADC value obtained within the tumour (**d**) was 1.462 × 10^−3^ mm²/s, which would also be considered to be of low (Gleason score 6) grade using previously reported thresholds. Comparing ADC values across systems is challenging due to a lack of standardization, and an ADC ratio has been previously proposed as a better metric to compare ADC. An ADC value from the contralateral normal PZ obtained at the same level was 2.000 × 10^−3^ mm²/s, which yields an ADC ratio of 0.73, which would be compatible with a Gleason score ≥7 tumour based on previously published thresholds. Corresponding DCE image (**e**) from the same level demonstrated a focal enhancing nodule with a type III contrast curve. Based on the imaging findings, a repeat TRUS-guided biopsy was performed, which demonstrated Gleason pattern 4 in the left apical PZ

DWI improves the detection of cancer foci in the PZ and TZ, and ADC values derived from DWI have been repeatedly shown to inversely correlate with Gleason grade of PCa [18, 38]. DWI is the single best imaging test for analysis of the PZ and is used in conjunction with T2W MRI for evaluation of TZ lesions [58, 62]. The visual analysis of DWI must include assessment of trace EPI and should not rely solely upon review of the derived apparent diffusion coefficient (ADC) map. Structures that are inherently of low T2W SI will appear dark on the ADC map because of an inherently low T2W SI (T2 “black-hole” effect) and not from true restricted diffusion (Figs. 1 and 2). Review of trace EPI is critical to avoid this common pitfall because these structures will not appear bright on long (b ≥1000 mm²/s) EPI (Figs. 1 and 2). Conversely, PCa (and as discussed some stromal BPH nodules) is of low SI on ADC and will appear bright on the long (b ≥1000 mm²/s) EPI (Figs. 3, 6 and 7). The use of longer b values (b ≥1000 mm²/s) facilitates the detection of cancer foci compared to the benign prostatic tissues [63, 64]. Visual analysis of ADC may be further limited by automatic display settings and can result in the failure to detect PCa or an underestimation of tumour grade if not displayed properly (Fig. 13). A previous study demonstrated that by using a window width = 1.650 and level = 1.675 × 10–6 mm²/s for display of ADC, higher grade tumours were more likely to appear dark (Fig. 13) [65].

It has been demonstrated repeatedly that ADC values inversely correlate to PCa grade [18]. The use of ADC values across MR systems is problematic because of differences related to magnet strength, vendor system and selection of differing b values [57]. Furthermore, a variety of methods to quantify ADC have been described including single region of interest measurement versus whole lesion analysis and mean, median and complex histogram analysis of ADC. Application of quantitative ADC thresholds may be limited by these factors. A recent study demonstrated that the use of an ADC ratio (normalised to adjacent normal PZ) provided a better inter-scanner comparison than the absolute ADC value [57]. Mean ADC values may also be limited due to the variable composition of prostate cancers and when comparing intermediate prostate cancers (Gleason score 3 + 4 = 7 vs. 4 + 3 = 7) [66] and studies have demonstrated that the use of the low percentile ADC may be a better reflector of the Gleason score [67, 68]. Regardless of the method used, due to differences across systems, at present, we suggest that the use of quantitative ADC in clinical practice should be applied carefully, derived and validated on an institutional basis.

(3) **DCE lacks standardisation and is limited in the TZ**

DCE improves the detection of PCa and DCE parameters correlate with PCa grade [69–71]. Currently, there is no established interpretation criteria for DCE analysis which varies from simple visual analysis to semi-quantitative analysis to full quantitative pharmacokinetic modelling [72]. A recent study demonstrated that DCE is underutilised in clinical practice compared to DWI and that semi-quantitative and quantitative analyses were not commonly used [72]. In the revised PI-RADS v2.0 guidelines, DCE analysis has been modified from semi-quantitative curve analysis to a simple visual analysis [58].

Moreover, DCE is very limited for the detection and grading of cancers in the transition zone because of significant overlap with stromal BPH (Fig. 7). Analysis of potential tumour foci in the transition zone should preferentially favour T2W and DWI over DCE. It is currently recognised that the utility of DCE in clinical practice is mainly as a confirmatory sequence for PZ lesions [58]; however, further studies are required to determine the value of DCE in PCa.

(4) **Targeted biopsy of MR-detected lesions using TRUS-guidance is challenging**

MP-MRI for PCa has transformed practice, but has created new challenges. Obtaining accurate histological correlation from lesions detected at MP-MRI may be challenging. MRI guided biopsy is accurate; however, this technique is limited by cost, availability of and access to MRI, prolonged procedure times and patient discomfort [8]. The use of TRUS guidance for biopsy of MP-MRI-detected lesions is currently a preferred option.

**Fig. 14:**
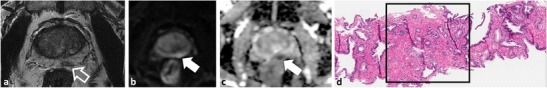
A 59-year-old patient with low-volume Gleason score 3 + 3 = 6 at non-targeted TRUS biopsy underwent MP-MRI to exclude clinically significant cancer after an increase in PSA. Axial T2W TSE image (**a**) demonstrates a low T2 SI focus in the left PZ (*open white arrow*) with restricted diffusion; note increased SI on trace b1000 mm²/s EPI and low SI on ADC (white arrows in **b** and **c**, respectively). Based on the MRI and clinical findings a diagnosis of potential higher grade tumour was suggested and a targeted repeat biopsy was performed. At time of repeat biopsy, which used cognitive registration of MP-MRI and TRUS data, no lesion could be identified at TRUS. Only one core biopsy through the left medial mid peripheral zone sextant was performed. Microscopic image from repeat TRUS-guided biopsy (**d**) demonstrates Gleason pattern 3 tumour. Continued in Fig. 14

**Fig. 15:**
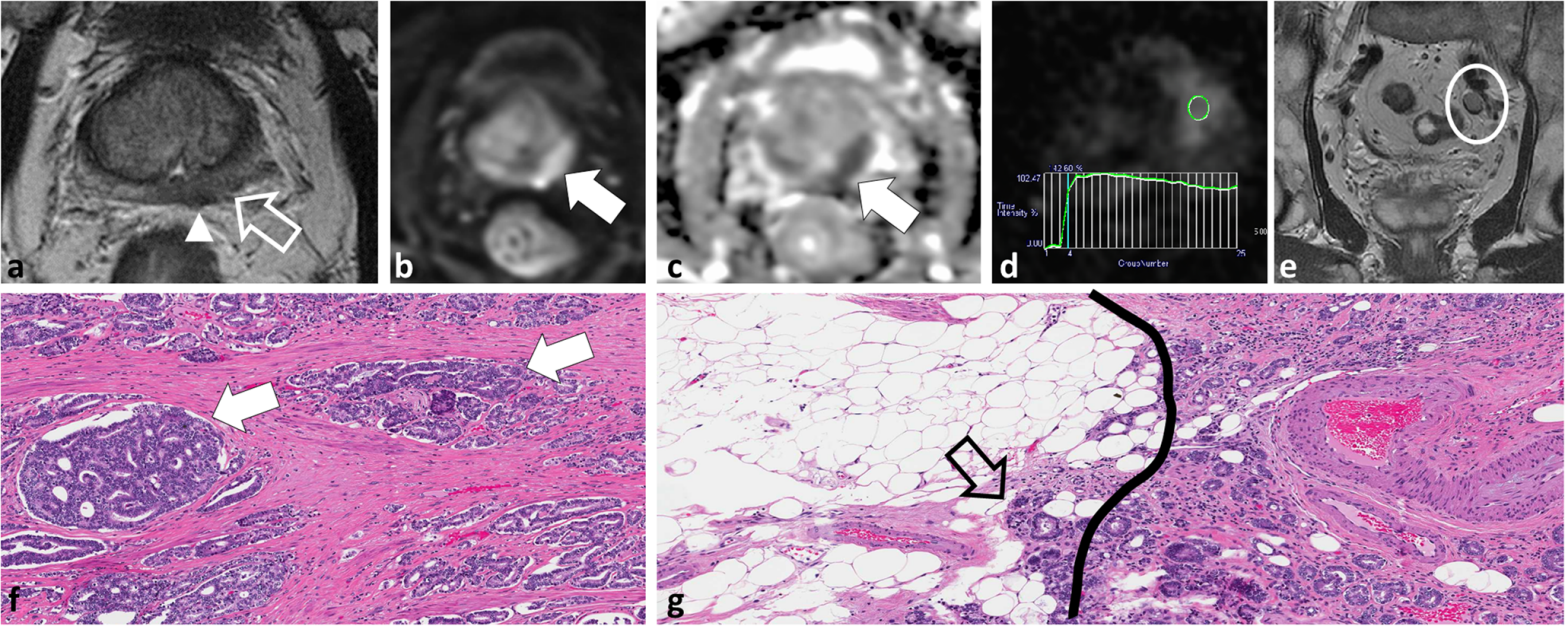
Although only low-volume Gleason score 3 + 3 = 6 was again noted at repeat TRUS biopsy (Fig. 13), a follow-up MP-MRI was performed 3 months later because of interval doubling of PSA to re-evaluate for a focus of higher grade cancer. Axial T2W TSE image (**a**) demonstrates that the small low T2 SI focus in the left PZ has grown substantially (*open white arrow*) with bulging and nodular extension into the peri-prostatic fat (*arrowhead*), which was reported as representing extra-prostatic extension. The lesion again demonstrates restricted diffusion; note increased SI on trace b1000 mm²/s EPI and low SI on ADC (*white arrows* in **b** and **c**, respectively) and demonstrated an aggressive type III contrast curve on DCE (**d**). In the interval, a malignant-appearing lymph node developed along the left pelvic sidewall (**e**). Based on these findings a diagnosis of high-grade tumour with extra-prostatic extension and metastatic adenopathy was provided. The patient underwent RP based on the imaging findings, he declined a repeat biopsy because of previous urosepsis related to prior TRUS biopsy. Corresponding microscopic images (**f** and **g**) demonstrate Gleason pattern 4 tumour (*white arrows* in **f**) and extra-prostatic extension of tumour (*open arrow*), which is beyond the prostate capsule (*black line*) and into the peri-prostatic fat (**g**)

**Fig. 16:**
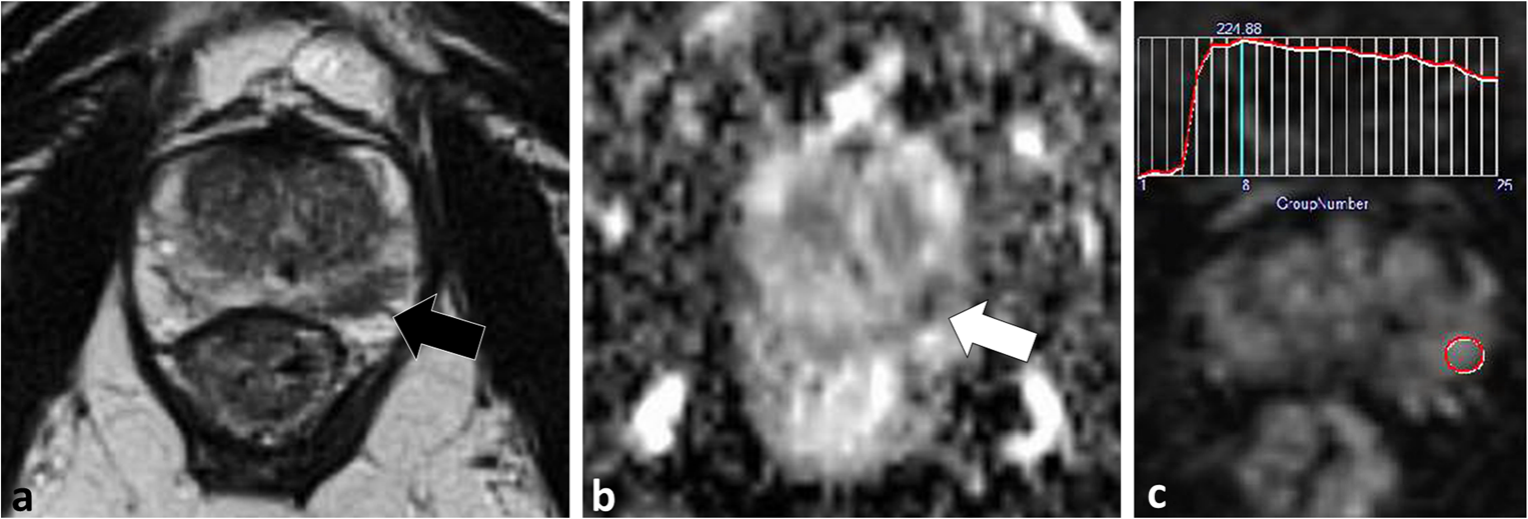
A 63-year-old patient underwent MP-MRI prior to routine repeat biopsies as part of his active surveillance protocol. Axial T2W TSE (**a**), axial ADC map (**b**) and semi-quantitative contrast curve derived from DCE (**c**) demonstrate a focal low T2W SI lesion (*black arrow*) with restricted diffusion (*white arrow*) and type III kinetics in the left middle peripheral zone. At repeat targeted biopsy using cognitive registration, no corresponding lesion could be identified. With a priori knowledge of the location of the lesion at MP-MRI, the TRUS operator performed three core needle biopsies through the left middle lateral and two core needle biopsies through the left middle medial PZ sextants. Results after targeted biopsies were Gleason 4 + 3 = 7 tumour with two out of three core biopsies in the left middle lateral PZ sextant

Using existing technology, so-called cognitive registration (CR) is performed. CR requires the TRUS operator to mentally integrate MP-MRI with real-time TRUS, identify suspicious MR lesions and, with TRUS guidance, target those lesions for biopsy. Published data regarding the applicability of CR are limited to institutional series and are highly subject to operator experience [8]. TRUS may be limited for the detection of tumours in the PZ and is also limited for assessment of the TZ and the anterior gland in many patients with enlarged glands due to poor ultrasound beam penetration. A failure to recognise the limitations of TRUS when performing CR biopsies can result in a failure to adequately sample suspicious areas on MP-MRI and ultimately result in delayed diagnosis and therapy for clinically significant PCa (Figs. 14 and 15). In our experience, cognitively registered TRUS biopsy of MP-MRI-detected lesions can be successful, provided that the TRUS operator recognises the limitations of TRUS for demonstrating a corresponding lesion to that seen on MP-MRI. It is not uncommon for a lesion detected in the PZ or TZ on mp-MRI to be sonographically occult on TRUS and, in these instances, by oversampling the corresponding location using anatomic landmarks, the diagnostic yield can be significantly improved (Fig. 16).

The use of Fusion software (which automatically integrates MP-MRI data with real-time 3D TRUS images) provides an alternative to cognitive registration [8]. Fusion software is not without its own limitations including mainly errors in fusion that relate to the spatial deformation of the prostate at TRUS compared to mp-MRI [8]. Moreover, this technology is expensive and at the moment is available in a few specialised centres, although availability is increasing. Studies comparing cognitively registered targeted TRUS biopsy to software fused targeted TRUS biopsy are lacking and have shown mixed results. While several studies have shown no difference between CR and Fusion software [73, 74], other studies have shown an improvement in the detection rate of cancer using fusion software systems [75, 76].

It is critical to emphasise that when a targeted TRUS-guided biopsy performed for a suspicious lesion detected on MP-MRI (using either cognitive registration or fusion software) is negative, the MP-MRI should be reviewed in the context of the biopsy results and other clinical factors in order to consider the possibility of an erroneous targeted biopsy. In these instances, repeat MRI or targeted biopsies can be contemplated.

## Conclusion

In conclusion, MP-MRI has become a critical component for patients being considered for or enrolled in active surveillance protocols for the management of low-grade and low-volume prostate cancers. MP-MRI is a proven imaging modality that can detect clinically significant foci of prostate cancer with high degrees of accuracy; the high negative predictive value of MP-MRI is particularly well suited for the AS of PCa. A number of pitfalls, both interpretive and technical, may be encountered at MP-MRI of the prostate and a failure to recognise these pitfalls in the AS population can result in suboptimal patient care. Targeted biopsies of MP-MR-detected lesions poses a new challenge and opportunity in clinical practice. The limitations of TRUS-guidance for lesion detection during targeted biopsies should be acknowledged in order to improve the diagnostic yield of targeted biopsies. A thorough understanding of these MP-MRI pitfalls is important for the MR practitioner involved in the management of prostate cancer.
